# Three-dimensional analysis of the tricuspid annular geometry in healthy subjects and in patients with different grades of functional tricuspid regurgitation

**DOI:** 10.1186/s12947-023-00315-7

**Published:** 2023-09-15

**Authors:** Gintarė Bieliauskienė, Ieva Kažukauskienė, Rita Kramena, Aleksejus Zorinas, Antanas Mainelis, Diana Zakarkaitė

**Affiliations:** 1 Clinic of Cardiovascular Diseases, Institute of Clinical Medicine, Faculty of Medicine, Vilnius University, M. K. Čiurlionio 21, 03101 Vilnius, Lithuania; 2 Department of Pathology, Forensic Medicine, Faculty of Medicine, Vilnius University, M. K. Čiurlionio 21, 03101 Vilnius, Lithuania; 3 Center of Cardiology and Angiology, Vilnius University Hospital Santaros Klinikos, Santariskiu St. 2, 08661 Vilnius, Lithuania; 4 Faculty of Mathematics and Informatics, Vilnius University, Naugarduko 24, 03225 Vilnius, Lithuania

**Keywords:** Three-dimensional echocardiography, Tricuspid valve, Tricuspid regurgitation, Tricuspid annulus

## Abstract

**Background:**

Accurate sizing of the tricuspid valve annulus is essential for determining the optimal timing of tricuspid valve (TV) intervention. Two-dimensional (2D) echocardiography has limitations for comprehensive TV analysis. Three-dimensional (3D) imaging of the valve provides a better understanding of its spatial anatomy and enables more accurate measurements of TV structures.

**Objectives:**

The study aimed to analyze tricuspid annulus (TA) parameters in normal heart and in different grades of functional tricuspid regurgitation (TR); to compare TA measurements obtained by 2D and 3D echocardiography.

**Methods:**

One hundred fifty-five patients (median age 65 years, 57% women) with normal TV and different functional TR grades underwent 2D and 3D transthoracic echocardiography. The severity of TR was estimated using multiparametric assessment according to the guidelines. Mid-systolic 3D TA parameters were calculated using TV dedicated software. The conventional 2D systolic TA measurements in a standard four-chamber view were performed.

**Results:**

In mid-systole, the normal TA area was 9.2 ± 2.0 cm^2^ for men and 7.4 ± 1.6 cm^2^ for women. When indexed to body surface area (BSA), there were no significant differences in the 3D parameters between genders. The 2D TA diameters were smaller than those measured in 3D. The ROC curve analysis identified that all 3D TA parameters can accurately differentiate between different functional TR grades. Additionally, the optimal cut-off values were identified for each TA parameter.

**Conclusions:**

Gender, body size, and age have an impact on the TA parameters in healthy subjects. 2D measurements are smaller than 3D parameters. The reference values for 3D metrics according to TR severity can help in identifying TA dilation and distinguishing between different functional TR grades.

**Graphical Abstract:**

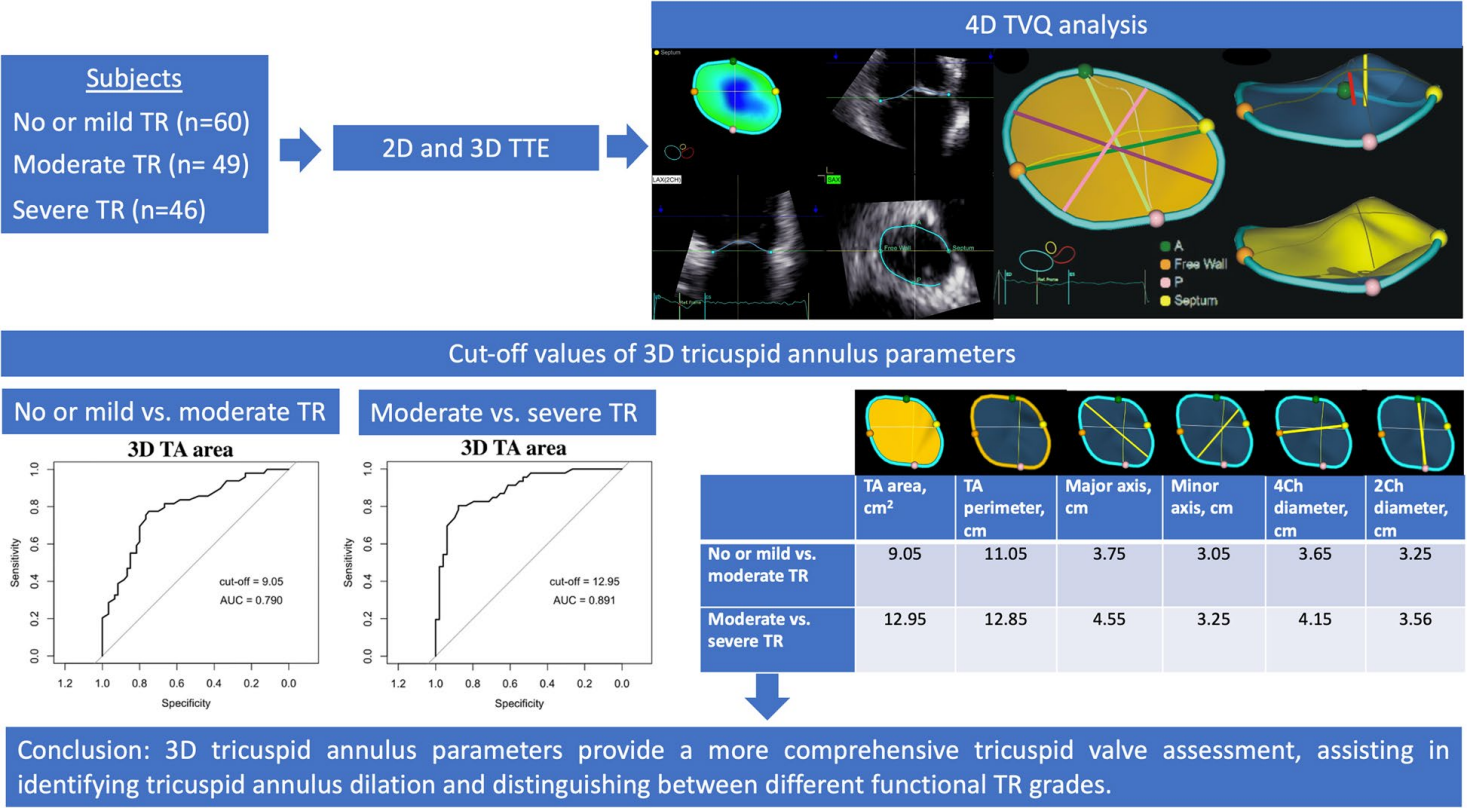

**Supplementary Information:**

The online version contains supplementary material available at 10.1186/s12947-023-00315-7.

## Introduction

A normal tricuspid valve (TV) has a complex spatial saddle-shaped morphology [1, 2]. The annulus is a dynamic structure that changes throughout the cardiac cycle and reaches its minimum size in mid-to-late systole [3]. With the progression of the functional tricuspid regurgitation (TR), the TV anatomy changes: the annulus dilates, and becomes more planar and circular [4]. Accurate sizing of the TV is crucial for determining the need for TV interventions and the choice of surgical technique. However, comprehensive TV assessment remains challenging.

In daily practice, we assess tricuspid annulus (TA) dilation from the two-dimensional (2D) echocardiography four chambers (4Ch) apical view. TV annulus is considered dilated when the TA diameter is ≥ 40 mm (or ≥ 21 mm/m^2^) [5]. However, the analysis of the TV using 2D echocardiography is limited by the nonplanar geometry of the TV. A thorough evaluation of the TV using three-dimensional (3D) echocardiography provides a better understanding of TV geometry and more accurate TA size determination. Nevertheless, there are still no normal 3D TA values in the current guidelines.

For a long time, there was no appropriate tool for 3D TV analysis. Recently, a new software dedicated to TV was developed and became commercially available. This new tool facilitated 3D TV analysis, making it less time-consuming and more user-friendly. Further studies are needed to evaluate 3D TV anatomy and its detailed changes in various pathologies.

The purpose of this study was to: (1) assess the impact of sex, body surface area (BSA), and age on normal TA anatomy; (2) compare TA measurements obtained using 2D and 3D echocardiography; and (3) determine the TA area, TA area indexed to BSA, TA perimeter, TA major axis, and TA major axis indexed to BSA thresholds for different grades of functional TR; (4) compare 3D TV parameters in patients with AF-TR and non AF-TR.

## Materials and methods

### Study population

Vilnius Regional Biomedical Research Ethics Committee has granted approval for this study (protocol number 2019/6–1131-630). From June 2021 to April 2022, a total of 186 individuals, prospectively underwent 2D and 3D transthoracic echocardiography in Vilnius University Hospital Santaros Klinikos, Vilnius, Lithuania. All participants provided a written consent form to take part in the study. Inclusion criteria for patients with no or mild TR were as follows: age ≥ 18 years, no prior history of cardiovascular or lung disease, absence of symptoms or medications, normal 2D echocardiography (defined as normal LV, RV and atrial size and function with left ventricular ejection fraction > 55%), and no evidence of valvular heart disease. For the cohort with functional moderate or severe TR, the inclusion criteria were: age ≥ 18 years, functional moderate or severe TR defined according to the guidelines [6, 7].

The exclusion criteria for the study included suboptimal quality of echocardiographic images. A total of 186 patients were enrolled in the study, and 31 were excluded due to poor-quality echocardiographic images. The remaining 155 patient were included in the statistical analysis.

Two criteria were used to group patients: (i) TR grade, and (ii) functional TR phenotype (AF-TR or non AF-TR). In scenario (i), all 155 patients were distributed into three groups based on TR severity: no or mild TR (*n* = 60), moderate TR (*n* = 49), and severe TR (*n* = 46).

In scenario (ii), 95 patients with moderate and severe TR were classified into two groups: AF-TR group (*n* = 63), patients with the absence of any TR cause other than persistent/permanent atrial fibrillation, and non AF-TR group (*n* = 32), the rest of the patients (when the coexisting pathological conditions occurred).

### Image acquisition

2D and 3D images were acquired using the commercially available Vivid E95 ultrasound machine (GE Healthcare; Horten, Norway) equipped with the 4Vc probe. Acquisition of 3D images of TV was performed from the right ventricular (RV)-focused apical view. To obtain an optimal view, the gain, sector width, and depth settings were adjusted. The R-wave-gated acquisition was performed over 4–6 beats during a single breath-hold. In patients with atrial fibrillation, single-beat narrow volume with minimal depth was used. For patients with TR, 3D echocardiography with color Doppler was performed.

### 2D echocardiography measurements

The TR severity was defined according to the guidelines of the American Society of Echocardiography [6] and the position paper on multi-modality imaging assessment of native valvular regurgitation of the European Association of Cardiovascular Imaging (EACVI) and European Society of Cardiology (ESC) [7]. Grading of TR severity was performed following current guidelines and using a multiparametric approach with a combination of qualitative (TV morphology, color flow TR jet, continuous-wave (CW) signal of TR jet) and semi-quantitative (hepatic vein flow, tricuspid inflow, proximal isovelocity surface area (PISA) radius, vena contracta (VC) width, 3D VC area) and quantitative (effective regurgitant orifice area (EROA), regurgitant volume (RVol)) assessment.

To assess RV function, the tricuspid annular plane systolic excursion (TAPSE) was obtained using M-mode imaging. Additionally, the right ventricular free wall longitudinal strain (FWS) analysis was performed using Automated Function Imaging (AFI) package (EchoPAC; GE Healthcare; Horten, Norway). Finally, the end-systolic TV annulus measurement in apical four chamber view (2D 4Ch diameter) was performed.

### 3D echocardiography image analysis

The measurements of TA were obtained using the 4D Auto TVQ Tricuspid Valve Quantification software package (EchoPAC; GE Healthcare; Horten, Norway). As TR mechanics is related to TV anatomy in mid-systole at maximal TR velocity, all TA parameters were measured in mid-systole [8, 9]. 4D Auto TVQ software provides the multiplanar reconstruction (MPR) of TV in 4-chamber (4Ch), 2-chamber (2Ch), and short axis views. Referential points were placed to mark the septum, free wall, anterior and posterior annulus, and leaflet coaptation points. The cardiac cycle timing (end-diastole and end-systole) was selected and manually adjusted if needed. The end-diastole was set as the first frame before TV closure, while the end-systole was set as the last frame before TV opening. The middle frame was selected between end-diastole and end-systole and considered mid-systole. Once the last point was marked, the segmentation process automatically began. If necessary, the TA landmarks were adjusted manually.

**Fig. 1:**
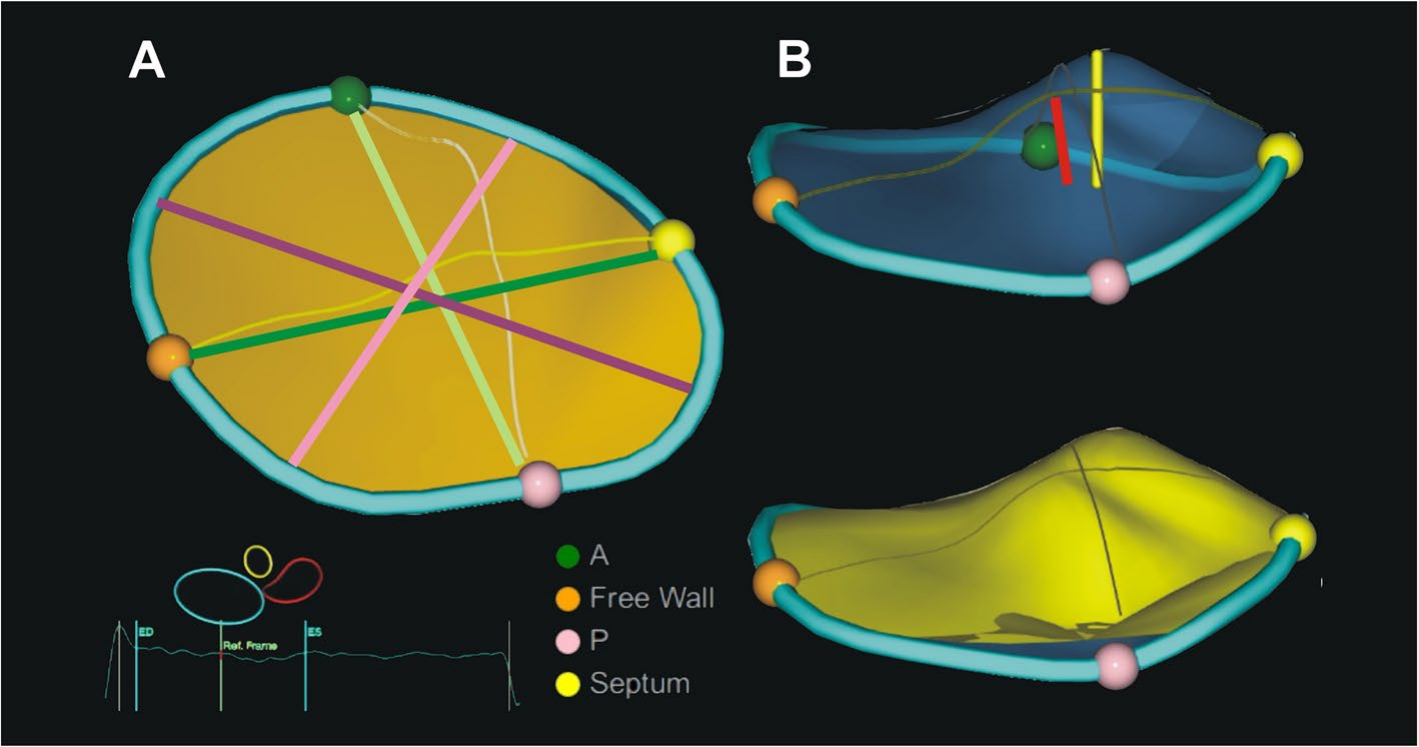
Tricuspid valve (TV) dimensions representing three-dimensional valve geometry at the referent frame in mid-systole. **A** dimensions of tricuspid annulus (TA): TA area- yellow area, TA perimeter- blue contour, 4Ch diameter- dark green line, 2Ch diameter- bright green light, major axis- dark pink line, minor axis- bright pink line. **B** dimensions of leaflet tethering: tenting volume- yellow surface, max tenting height- yellow line, coaptation point height- red line. Green, brown, purple, and yellow points marks are the anterior, free wall, posterior and septal edges of TA

The software provided measurements of 3D TA, as shown in Fig. 1, including TA area (the area of the non-planar surface delineated by the TA 3D contour); TA perimeter (the length of the 3D contour representing the TA circumference); 4Ch diameter (the maximal distance between septum and RV free wall derived from 3D contour); 2Ch diameter (the maximal distance between RV anterior and posterior wall derived from 3D contour); major axis (the longest diameter of the TA); minor axis (the shortest diameter of the TA); tenting volume (the volume between the leaflets and the TA surface); max tenting height (the peak distance of the valve surface to the TV plane); coaptation height (the height of the user-placed coaptation point to the 4Ch diameter of the TV annulus), and sphericity index (ratio between the minor and major axis of the TA) [10].

The parameters of 3D TA size were indexed to the body surface area (BSA). The reconstructed 3D echocardiography images with Color Doppler were used to measure the 3D vena contracta area [11].

### Reproducibility analysis

Interobserver and intraobserver variability were checked in a subset of 20 patients for all 3D TV measurements**,** including TA area, perimeter, major axis, minor axis, 4Ch diameter, 2Ch diameter, max tenting height, coaptation height, tenting volume, sphericity index. To determine intraobserver repeatability, a single observer measured the same parameter in repeated measurement. In addition, two independent observers analyzed the same image to determine interobserver variability.

### Statistical analysis

All statistical analysis was performed using R program package (version 4.0.4). Normally distributed continuous variables are presented as mean and standard deviation (SD). Other continuous variables are expressed as median with first and third quartiles. Categorical variables are presented as frequencies and percentage.

The Shapiro–Wilk test was used to assess the normality of quantitative variables. For comparisons between genders or two TR groups, either Student's t-test or the nonparametric Mann–Whitney U test was employed based on the appropriateness of the data distribution. Categorical variables were compared between the groups by the chi-square test or Fisher’s exact test if expected values were < 5. Pearson's or Spearman’s correlation was used to identify relationships between two quantitative variables as appropriate.

The receiver operating characteristic (ROC) curves were used to identify the best cut-off of TA parameters for different functional TR grades. Sensitivity and specificity for these cut-off values are also provided, along with the area under the curve (AUC) and its 95% confidence interval (CI).

Inter- and intra-rater reliability were assessed using the two-way random Intraclass Correlation Coefficient (ICC).

A *p*-value less than 0.05 was considered significant.

## Results

### Patients groups according to TR severity and their characteristics

The demographic characteristics, TA metrics, and the parameters of right chambers of the heart by TR grade are summarized in Table 1. No significant differences in sex distribution and body surface area were observed between the groups. However, patients with severe TR were significantly older than those with no or mild TR group. The severe TR group had a higher number of patients with atrial fibrillation compared to the moderate TR group. Meanwhile, pulmonary artery systolic pressure was similar in both moderate and severe TR groups.

**Table 1:** Demographics, tricuspid valve and right heart parameters in all three study groups

	**No or mild TR** **(*n* = 60)**	**Moderate TR** **(*n* = 49)**	*p*-value*	**Severe TR** **(*n* = 46)**	***p*-value†**
**Sex (female)**	30 (50)	30 (61.2)	0.241	28 (60.9)	0.972
**Age, yrs**	48.3 ± 16.6	64.4 ± 14.0	< 0.001	73.9 ± 10.2	< 0.001
**Weight, kg**	77.8 ± 15.6	77.7 ± 16.9	0.982	78.8 ± 16.4	0.752
**Height, cm**	173.7 ± 10.3	168.9 ± 10.5	0.022	166.3 ± 7.8	0.201
**BSA, m**^**2**^	1.9 ± 0.2	1.9 ± 0.2	0.536	1.9 ± 0.2	0.989
**Atrial fibrillation**	0 (0)	26 (46.9)	< 0.001	44 (95.7)	< 0.001
**PASP, mmHg**	-	41.8 ± 14.1	-	43.1 ± 10.1	0.648
**TA area, cm**^**2**^	8.3 ± 2.0	10.7 ± 2.3	< 0.001	15.2 ± 3.0	< 0.001
**TA perimeter, cm**	10.4 ± 1.3	11.8 ± 1.2	< 0.001	14.1 ± 1.4	< 0.001
**Major axis, cm**	3.6 ± 0.5	4.0 ± 0.5	< 0.001	4.7 ± 0.6	< 0.001
**Minor axis, cm**	2.8 ± 0.4	3.2 ± 0.5	< 0.001	3.9 ± 0.5	< 0.001
**4Ch diameter, cm**	3.0 ± 0.5	3.6 ± 0.5	< 0.001	4.4 ± 0.5	< 0.001
**2Ch diameter, cm**	3.0 ± 0.5	3.4 ± 0.5	< 0.001	4.0 ± 0.5	< 0.001
**Max tenting height, cm**	0.5 ± 0.2	0.7 ± 0.2	< 0.001	0.9 ± 0.3	< 0.001
**Coaptation height, cm**	0.4 ± 0.2	0.5 ± 0.2	< 0.001	0.7 ± 0.4	< 0.001
**Tenting volume, ml**	1.0 ± 0.6	1.7 ± 1.2	< 0.001	3.9 ± 2.3	< 0.001
**Sphericity index, %**	79.8 ± 9.1	79.6 ± 9.5	0.930	83.7 ± 8,7	0.031
**FWS, %**	-29.6 ± 3.9	-20.5 ± 7.0	< 0.001	-20.2 ± 5.7	0.830
**TAPSE, cm**	2.2 ± 0.4	1.5 ± 0.6	< 0.001	1.3 ± 0.4	0.053

Values are mean ± SD or n (%)

*BSA* body surface area, *4Ch* four chambers, *FWS* free wall strain, *PASP* pulmonary artery systolic pressure, *TAPSE* tricuspid annular plane systolic excursion, *TA* tricuspid annulus, *TR* tricuspid regurgitation, *2Ch* two chambers

^*^Comparison between no or mild TR and moderate TR

^†^Comparison between moderate TR and severe TR

**Fig. 2:**
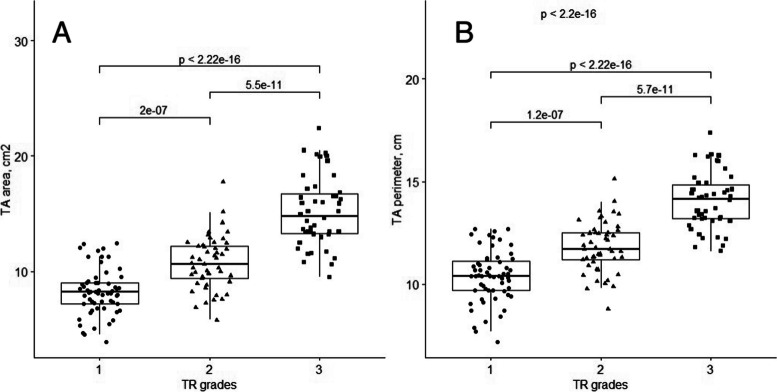
3D parameters of TA area (**A**) and perimeter (**B**) in different functional TR grades

Patients with moderate and severe TR had larger values for all 3D TV parameters (TA area, perimeter, 4Ch diameter, 2Ch diameter, major, minor axis, max tenting height, coaptation height, tenting volume) (Fig. 2), a higher sphericity index, and worse right ventricle systolic function (lower FWS and TAPSE) than the no or mild TR group.

### Interobserver and intraobserver variability in annular measurements

All 3D echocardiographic measurements showed optimal intra- and interobserver reproducibility (Table 2).

**Table 2:** Intra- and inter-observer variabilities in tricuspid valve 3D measurements

	**Intra-observer variability**		**Inter-observer variability**	
	**ICC**	***p*-value**	**ICC**	***p*-value**
**TA area, cm**^**2**^	0.95	< 0.001	0.97	< 0.001
**TA perimeter, cm**	0.94	< 0.001	0.96	< 0.001
**Major axis, cm**	0.94	< 0.001	0.96	< 0.001
**Minor axis, cm**	0.91	< 0.001	0.91	< 0.001
**4Ch diameter, cm**	0.93	< 0.001	0.95	< 0.001
**2Ch diameter, cm**	0.88	< 0.001	0.90	< 0.001
**Max tenting height, cm**	0.90	< 0.001	0.90	< 0.001
**Coaptation height, cm**	0.85	< 0.001	0.86	< 0.001
**Tenting volume, ml**	0.91	< 0.001	0.97	< 0.001
**Sphericity index, %**	0.90	< 0.001	0.92	< 0.001

*4Ch* four chambers, *ICC* intraclass correlation coefficient, *TA* tricuspid annulus, *3D* three-dimensional, *2Ch* two chambers

### Normal tricuspid valve anatomy

The TA area at mid-systole was 8.3 ± 2.0 cm^2^ in patients with no or mild TR. The major axis diameter was larger than the 4Ch diameter (3.6 ± 0.5 cm vs. 3.0 ± 0.5 cm, *p* < 0.001).

**Fig. 3:**
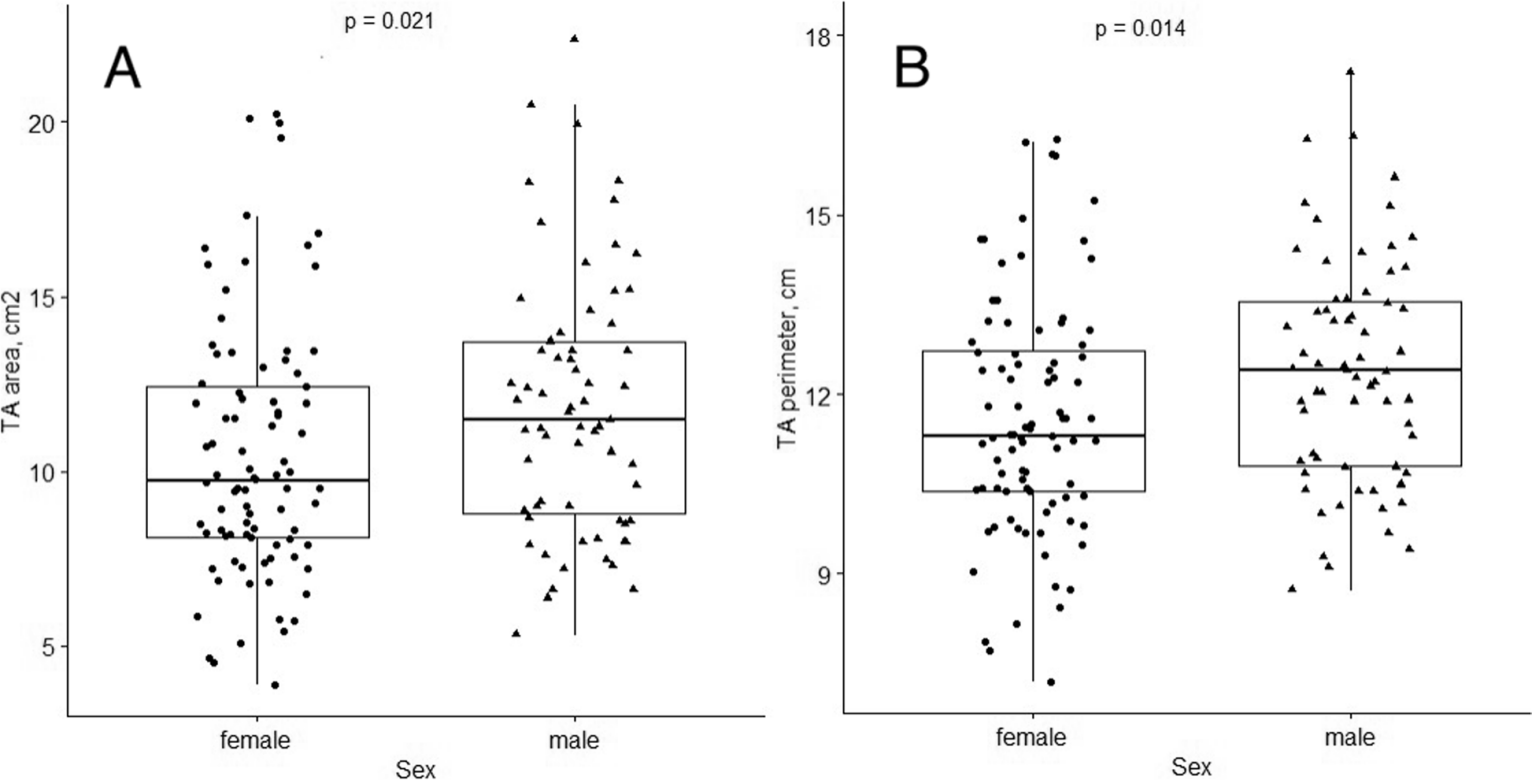
3D parameters of TA area (**A**) and perimeter (**B**) in different genders in no or mild TR group. The 3D parameters such as TA area, perimeter were larger in males than in females

The 3D parameters such as TA area, perimeter, 4Ch diameter, major, minor axis, and tenting volume were statistically significantly larger in males than in females (Fig. 3). However, when indexed to BSA, there was no statistically significant difference between genders (Table 3).

**Table 3:** Tricuspid annulus parameters in men and women in no or mild TR group

**Nonindexed**	**Men**	**Women**	***p* value**
**TA area, cm**^**2**^	9.2 ± 2.0	7.4 ± 1.6	< 0.001
**TA perimeter, cm**	11.0 ± 1.1	9.8 ± 1.1	< 0.001
**Major axis, cm**	3.6 ± 0.5	3.3 ± 0.4	< 0.001
**Minor axis, cm**	3.1 ± 0.4	2.6 ± 0.3	< 0.001
**4Ch diameter, cm**	3.3 ± 0.4	2.8 ± 0.5	< 0.001
**2Ch diameter, cm**	3.2 ± 0.5	2.9 ± 0.4	0.071
**Max tenting height, cm**	0.6 ± 0.1	0.5 ± 0.2	0.068
**Coaptation height, cm**	0.4 ± 0.2	0.3 ± 0.2	0.155
**Tenting volume, ml**	1.2 ± 0.6	0.8 ± 0.4	0.002
**Sphericity index, %**	81 ± 9	79 ± 9	0.362
**Indexed (to BSA)**	**Men**	**Women**	***p* value**
**TA area, cm**^**2**^**/m**^**2**^	4.5 ± 1.1	4.1 ± 1.0	0.125
**TA perimeter, cm/m**^**2**^	5.4 ± 0.8	5.5 ± 0.9	0.640
**Major axis, cm/m**^**2**^	1.9 ± 0.3	1.9 ± 0.3	0.994
**Minor axis, cm/m**^**2**^	1.5 ± 0.3	1.5 ± 0.2	0.397
**4Ch diameter, cm/m**^**2**^	1.6 ± 0.3	1.5 ± 0.3	0.125
**2Ch diameter, cm/m**^**2**^	1.6 ± 0.5	1.6 ± 0.6	0.220

Values are mean ± SD

*BSA* body surface area, *4Ch* four chambers, *TA* tricuspid annulus, *TR* tricuspid regurgitation, *2Ch* two chambers

4Ch diameter (*r* = 0.27, *p* < 0.039) and tenting volume (*r* = -0.47, *p* < 0.001) correlated with the age in this group. The younger patients had smaller 4Ch diameter, larger tenting volume, and lower sphericity index.

### Comparison between 2 and 3D echocardiography measurements

3D 4Ch diameter, obtained from 3D echocardiography dataset, moderately correlated with 2D 4Ch diameter, measured from 2D echocardiography apical four chamber view (*r* = 0.51, *p* < 0.001. Furthermore, 2D 4Ch diameter was smaller than the 3D 4Ch diameter (2.85 ± 0.4 cm vs. 3.00 ± 0.5 cm, *p* < 0.01, respectively). The 3D TA major axis, which represents the largest diameter of an ellipse that fits the TA shape, was significantly larger than 2D 4Ch diameter (3.6 ± 0.5 cm vs. 2.9 ± 0.4 cm, *p* < 0.001, respectively).

### Cut-off values of 3D TA parameters to differentiate different TR grades

**Fig. 4:**
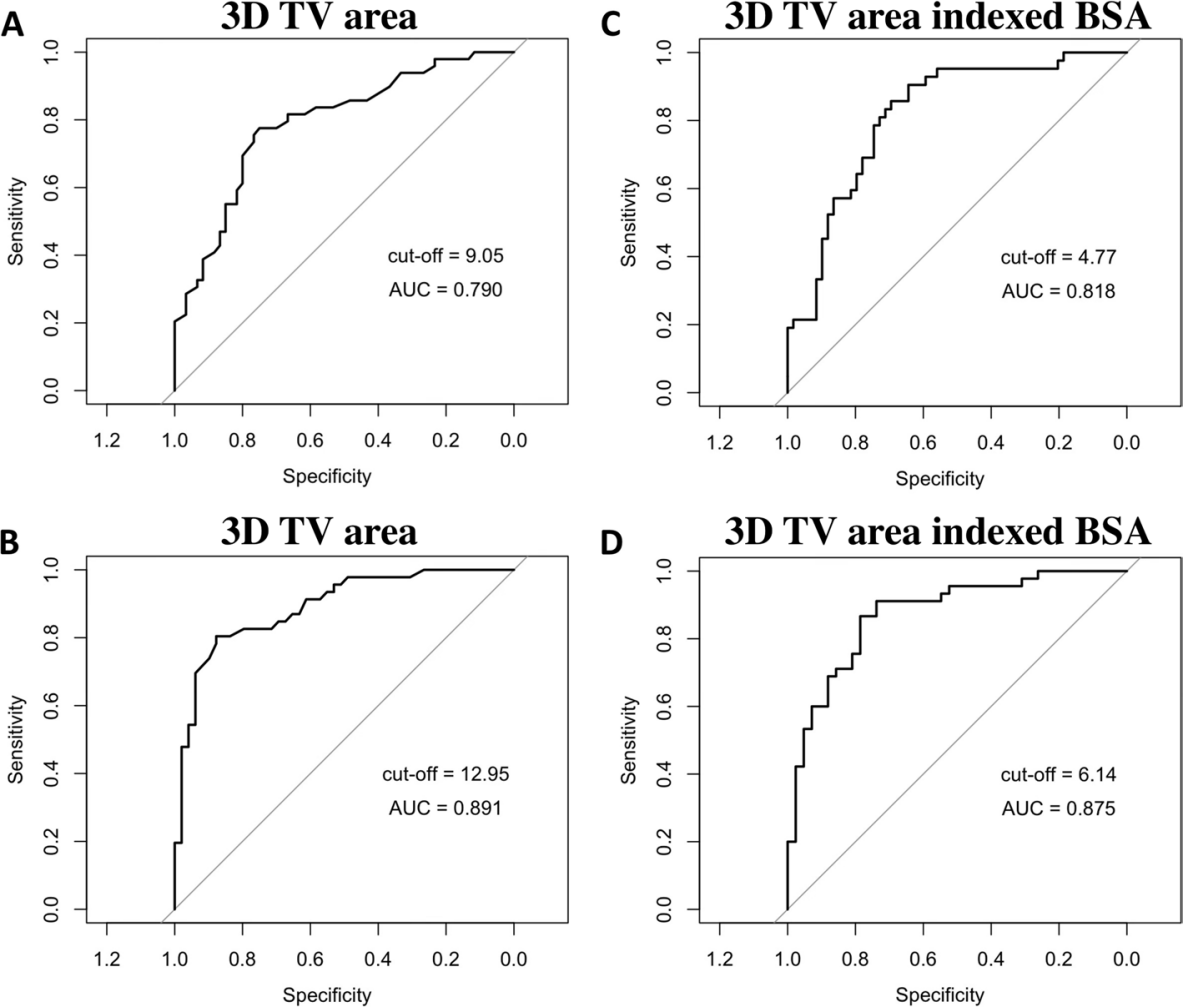
Receiver operating characteristics (ROC) curve analysis shows that: (**A**) 3D TV area with cut-off value of 9.05 cm^2^ distinguishes mild TR from moderate; (**B**) 3D TV area with the cut-off value of 12.95 cm^2^ distinguishes moderate TR from severe; (**C**) 3D TV area indexed BSA with the cut-off value of 4.77 cm^2^/m^2^ distinguishes mild TR from moderate; (**D**) 3D TV area indexed BSA with the cut-off value of 6.14 cm^2^/m^2^ distinguishes moderate TR from severe. AUC- area under the curve

**Fig. 5:**
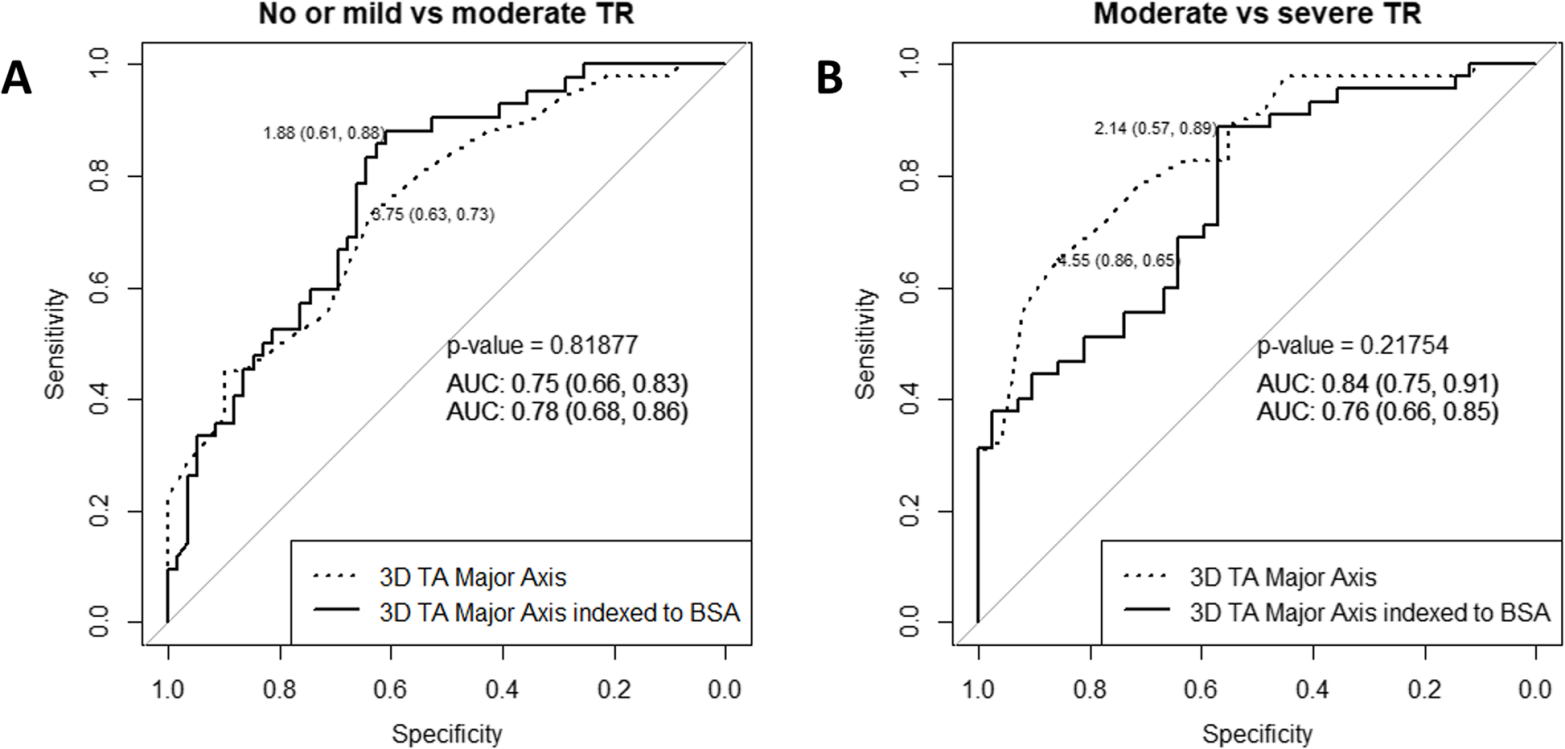
TA major axis and TA major axis indexed to BSA cut-off values to differentiate: (**A**) no or mild from moderate TR; (**B**) moderate from severe TR

We conducted a ROC analysis (Tables 4 and 5, Figs. 4 and 5) to assess the discriminative capabilities of 3D TA echocardiographic parameters in distinguishing between various degrees of TR severity. The analysis also aimed to identify optimal cut-off values (Youden index) for each parameter and its value indexed to BSA.

**Table 4:** ROC curves analysis of TV 3D geometry parameters to differentiate no or mild TR from moderate

	**AUC**	**95% CI**	**Cut-off**	**Specificity**	**Sensitivity**
**TA perimeter, cm**	0.80	0.70–0.87	11.05	0.73	0.78
**4ch diameter, cm**	0.80	0.72–0.88	3.65	0.93	0.51
**TA area, cm**^**2**^	0.79	0.70–0.87	9.05	0.75	0.78
**Major axis, cm**	0.75	0.66–0.84	3.75	0.63	0.73
**Minor axis, cm**	0.71	0.61–0.80	3.05	0.67	0.68
**Tenting volume, ml**	0.69	0.58–0.79	1.35	0.75	0.63
**2ch diameter, cm**	0.68	0.58–0.78	3.25	0.68	0.61
**Max tenting height, cm**	0.67	0.57–0.77	0.75	0.97	0.41
**Coaptation height, cm**	0.66	0.55–0.77	0.65	0.69	0.62
**Values indexed to BSA**					
**TA perimeter, cm**	0.77	0.68–0.86	5.50	0.58	0.90
**4ch diameter, cm**	0.83	0.75–0.91	1.64	0.66	0.95
**TA area, cm**^**2**^	0.82	0.73–0.90	4.77	0.69	0.86
**Major axis, cm**	0.78	0.68–0.86	1.88	0.61	0.88
**Minor axis, cm**	0.73	0.63–0.83	1.66	0.76	0.62
**2ch diameter, cm**	0.67	0.56–0.77	1.66	0.61	0.69

*AUC* area under the curve, *BSA* body surface area, *ROC* receiver operating characteristic, *CI* confidence interval, *TA* tricuspid annulus, *TR* tricuspid regurgitation, *TV* tricuspid valve, *3D* three dimensional, *VCA* vena contracta area, *EROA* effective regurgitant orifice area, *VC* vena contracta, *4Ch* four chambers, *2Ch* two chambers

**Table 5:** ROC curves analysis of TR and TV 3D geometry parameters to differentiate moderate TR from severe

	**AUC**	**95% CI**	**Cut-off**	**Specificity**	**Sensitivity**
**TA area, cm**^**2**^	0.90	0.83–0.95	12.95	0.90	0.81
**TA perimeter, cm**	0.90	0.83–0.95	12.85	0.83	0.83
**Minor axis, cm**	0.86	0.78–0.93	3.25	0.67	0.96
**Major axis, cm**	0.85	0.77–0.92	4.55	0.88	0.66
**4ch diameter, cm**	0.85	0.78–0.92	4.15	0.83	0.68
**Tenting volume, ml**	0.82	0.74–0.90	2.95	0.88	0.64
**2ch diameter, cm**	0.81	0.72–0.89	3.56	0.67	0.83
**Max tenting height, cm**	0.74	0.64–0.84	0.65	0.58	0.84
**Coaptation height, cm**	0.66	0.55–0.77	0.65	0.69	0.62
**Values indexed to BSA**					
**TA area, cm**^**2**^	0.87	0.79–0.94	6.14	0.79	0.87
**TA perimeter, cm**	0.80	0.70–0.88	6.55	0.67	0.80
**Minor axis, cm**	0.81	0.71–0.90	1.87	0.81	0.76
**Major axis, cm**	0.76	0.65–0.86	2.14	0.57	0.89
**4ch diameter, cm**	0.84	0.75–0.91	1.99	0.74	0.84
**2ch diameter, cm**	0.74	0.63–0.84	1.79	0.57	0.89

*AUC* area under the curve, *BSA* body surface area, *ROC* receiver operating characteristic, *CI* confidence interval, *TA* tricuspid annulus, *TR* tricuspid regurgitation, *TV* tricuspid valve, *3D* three dimensional, *VCA* vena contracta area, *EROA* effective regurgitant orifice area, *VC* vena contracta, *4Ch* four chambers, *2Ch* two chambers

Our findings revealed that TA parameters exhibited remarkable discriminatory abilities in differentiating no or mild TR from moderate TR (Table 4). Notably, TA area, perimeter, and 4Ch diameter displayed very good AUC values of 0.79, 0.80, and 0.80, respectively. Additionally, when indexed to BSA, TA area and 4Ch diameter yielded AUC values of 0.82 and 0.83, respectively. According to the optimal cut-off points derived from the ROC analysis, when the TA area is less than 9.05 cm^2^ (sensitivity 0.78, specificity 0.75, *p* < 0.001), and the TA area indexed to BSA is less than 4.77 cm^2^/m^2^ (sensitivity 0.86, specificity 0.69, *p* < 0.001), we may expect to have no or mild TR. Additionally, a TA perimeter less than 11.05 cm (sensitivity 0,78 and specificity 0.73, *p* < 0.001), and a 4Ch diameter of 3.65 cm (sensitivity 0.51, specificity 0.93, *p* < 0.001) indicate no or mild TR.

Moreover, the performance of the TA parameters in distinguishing moderate TR from severe TR was even more pronounced (Table 5). TA area, perimeter, minor and major displayed notably high AUC values of 0.90, 0.90, 0.86 and 0.85, respectively. When indexed to BSA, TA area and perimeter maintained excellent discriminative ability, with AUC values of 0.87 and 0.80, respectively. Based on ROC analysis, when the TA area is greater than 12.95 cm^2^ (sensitivity 0.81 and specificity 0.90, *p* < 0.001), and the TA area indexed to BSA is greater than 6.14 cm^2^/m^2^ (sensitivity 0.87, specificity 0.79, *p* < 0.001), the TR is likely to be severe. Additionally, a TA perimeter greater than 12.85 cm (sensitivity 0.83 and specificity 0.83, *p* < 0.001), and a major axis greater than 4.55 cm (sensitivity 0.66 and specificity 0.88, *p* < 0.001) indicate severe TR.

### Comparison of 3D tricuspid valve geometry between AF-TR and non AF-TR

All parameters of the 3D TA geometry and regurgitation in both groups are listed in Supplement Table S1. The AF-TR and non AF-TR groups were similar with respect to age and gender. The distribution of TR severity grading was different between the AF-TR and non AF-TR patients: the majority of the patients had severe TR (*n* = 39; 61.9%) in the AF-TR group, and moderate TR (*n* = 24; 75%) in the non AF-TR group. Compared with non AF-TR, the AF-TR group showed a larger TA area, perimeter, major axis, 4Ch diameter, and tenting volume, whereas, PASP (pulmonary artery systolic pressure) was lower. The groups had no significant differences in max tenting height, coaptation height, sphericity index, and FWS.

**Fig. 6:**
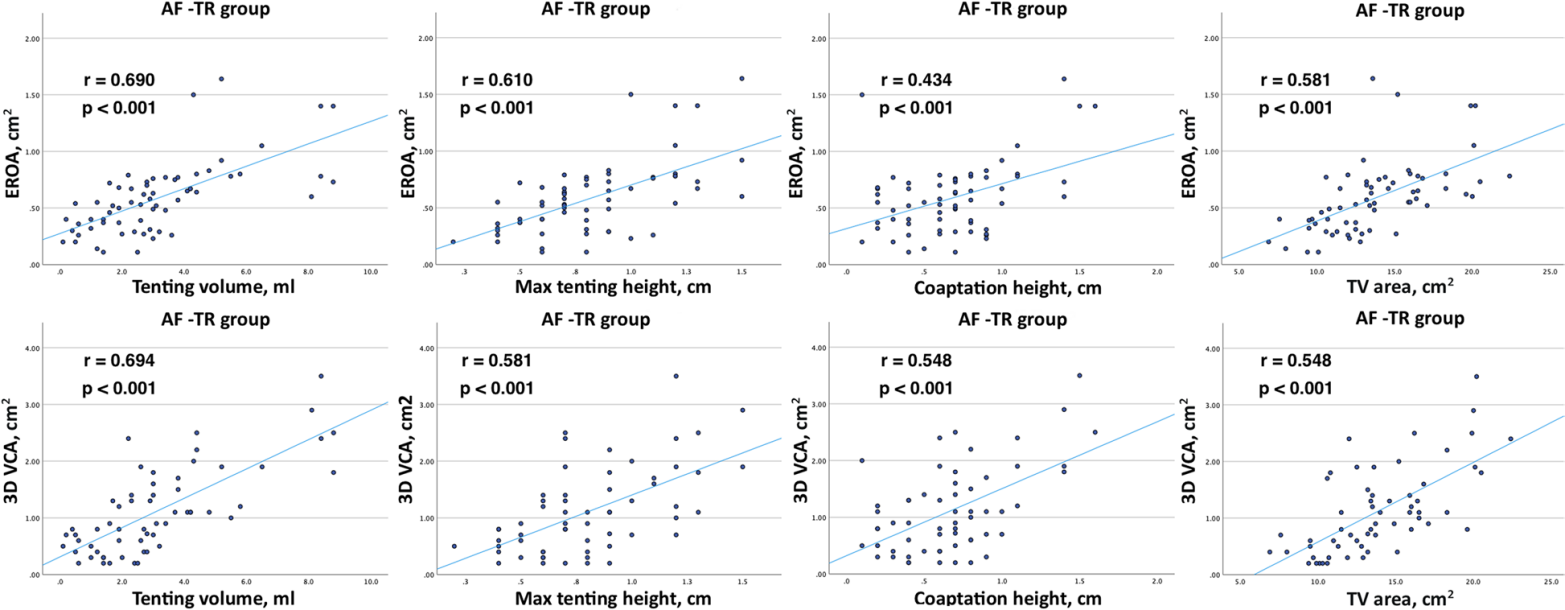
Correlation between TR severity parameters (EROA, 3D VCA) and TV geometry parameters (tenting volume, max tenting height, coaptation height, 3D TV area) in AF-TR group

**Fig. 7:**
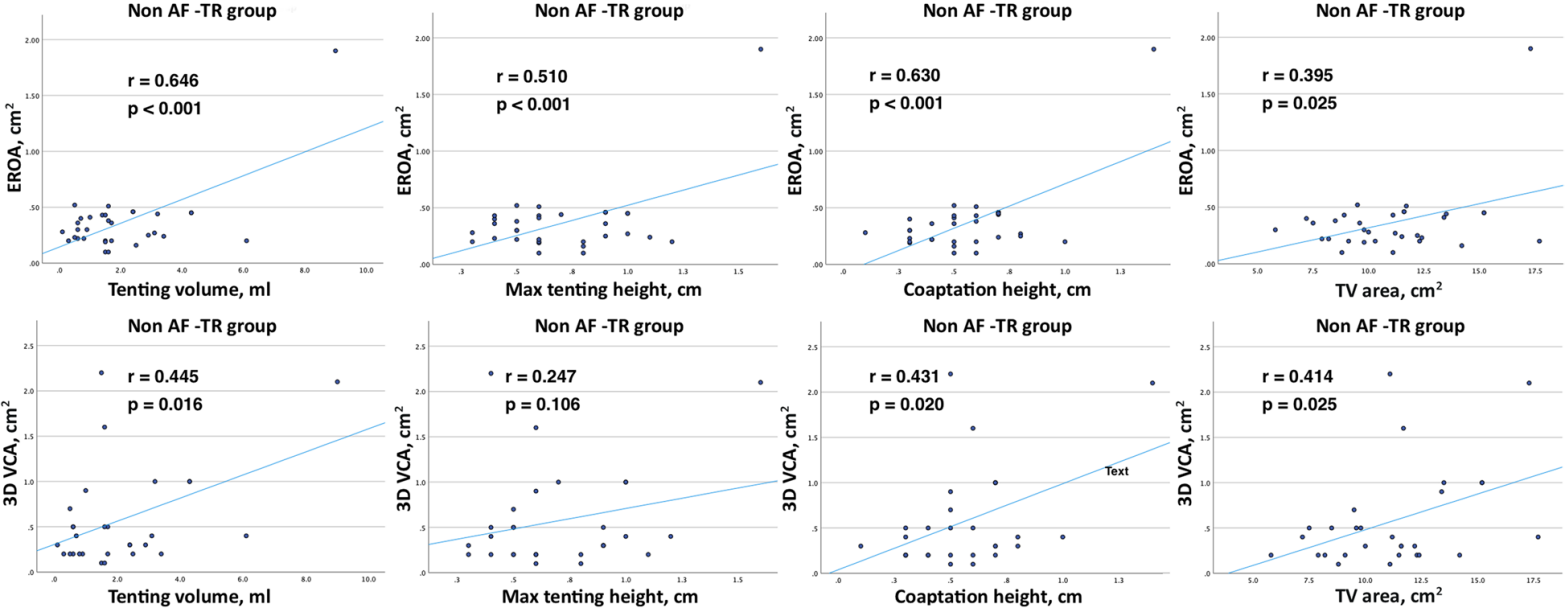
Correlation between TR severity parameters (EROA, 3D VCA) and TV geometry parameters (tenting volume, max tenting height, coaptation height, 3D TV area) in non AF-TR group

The correlation analysis between TR severity parameters and TV geometry parameters is shown in Fig. 6 and Fig. 7. The strongest correlations were found in AF-TR group between EROA and tenting volume, and 3D VCA and tenting volume (*r* = 0.690, *p* < 0.001 vs. *r* = 0.694, *p* < 0.001, respectively). A similar correlation was between EROA and max tenting height, and 3D VCA and max tenting height (*r* = 0.610, *p* < 0.001 vs. *r* = 0.581, *p* < 0.001, respectively). In addition, there was a strong correlation between EROA and TA area, and 3D VCA and TA area (*r* = 0.581, *p* < 0.001 vs. *r* = 0.656, *p* < 0.001, respectively). These correlations were lower but also significant in non AF-TR group.

## Discussion

This prospective study analyzed the 3D TA anatomy in healthy individuals and in patients with different grades of functional TR. The main findings of this study can be summarized as follows: (1) men had larger 3D TA parameters than women in normal heart, but there was no difference in 3D parameters between the genders when indexed to BSA; (2) younger patients had smaller 4Ch diameter, larger tenting volume, and lower sphericity index; (3) 2D parameters were smaller than 3D measurements; (4) 3D TA measurements provide a high diagnostic value for differentiation of the TR grades a with good sensitivity and specificity; (5) TA area, perimeter, major axis, 4Ch diameter, tenting volume were higher in AF-TR compared to non AF-TR.

### 3D analysis of the tricuspid valve in a normal heart

In our study, the 3D TA measurements were performed using a software dedicated specifically for the TV. Our study demonstrated results similar to those reported by other investigators, including D. Muraru [12] and K. Addetia et al. [3]. K. Addetia analyzed 3D TA dimensions in 209 healthy individuals throughout the cardiac cycle using the multiplanar reconstruction (MPR) method and custom software. The study by D. Muraru et al. was the first to assess the TV reference values and its dynamics during the cardiac cycle in 254 healthy volunteers using a commercially available 3D software package dedicated to the TV [12]. Both studies reported sex- and age-related differences and indexation for BSA.

Our analysis of the no or mild TR group, 3D TA parameters demonstrated gender differences, consistent with K. Addetia [3] and D. Muraru [12]. However, these differences disappeared after BSA indexation, aligning with the findings of K. Addetia [3] and D. Muraru [12], who reported persistent gender differences only for 3D TA area and 4Ch diameter, with larger TV perimeter and long-axis dimensions in women when indexed to BSA [3].

**Fig. 8:**
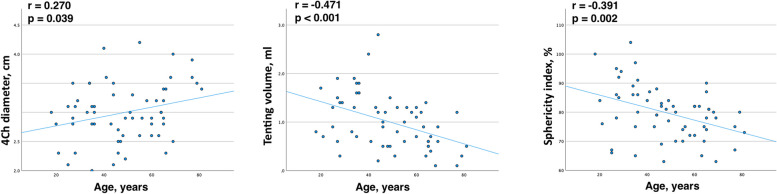
Correlation between TV parameters (4Ch diameter, tenting volume, sphericity index) and age in no or mild TR group

In our no or mild TR group, we did not find age-related correlations with TA area or perimeter, aligning with findings from K. Addetia et al. [3] and D. Muraru et al. [12], who also reported no significant age-related difference in TA area and perimeter. However, we did observed some age-related TA anatomical changes: 4Ch diameter increased with age, while, tenting volume and sphericity index decreased (Fig. 8). Younger patients in the no or mild TR group had greater tenting volume and lower sphericity index. Considering that clinically significant TR increases with age [11–14], further research is needed to investigate the impact of age on TA anatomical changes.

The 2D and 3D parameters comparison showed that the 2D 4Ch diameter was smaller than the 3D diameters, such as 4Ch diameter obtained from 3D echocardiography and major axis. These current findings may be supported by many previous studies [2, 3, 12, 15] that 2D echocardiography underestimates TV dimensions compared to 3D echocardiography. Therefore, 2D measurements are insufficient for evaluating the maximum diameter of the TA. Solely relying on 2D TA evaluation could be misleading and does not accurately reflect the degree of TA enlargement.

The timing during the cardiac cycle influences TA diameter [3]. In our study, 3D measurements were performed during mid-systole, while 2D 4Ch diameter was measured at the end-systole. Although there is a minimal difference between mid-systole and end-systole [3], the discrepancy between the 3D and 2D measurements could also be attributed to the differences in the timing of these measurements”.

### Cut-off values of TA parameters for different TR grade

The current guidelines only recommend using one metric, the 2D diameter in 4Ch apical view, to determine TA dilation. However, relying solely on a linear dimension cannot fully capture the geometry and size of a spatial structure. Therefore, it is necessary to incorporate additional parameters for a comprehensive assessment of TA dilation and to better understand the relationship between TA dimensions and the severity of functional TR. Studies [16, 17] suggest that 3D echocardiographic parameters can help in predicting the severity of TR. The size of the TA area directly correlates with functional TR severity [18] and increases with progressing functional TR [4]. Therefore, we sought to identify the reference values of 3D TA dimensions.

Our study is the first to provide the cut-off values of the TA parameters for different functional TR grades. While significant differences were observed among all the TA parameters in their ability to differentiate between different grades of TR severity, it is noteworthy that the parameters tended to achieve higher discriminative accuracy when distinguishing moderate TR from severe TR compared to differentiating no or mild TR from moderate TR. The AUC values for the TA area and major axis, when indexed to BSA, demonstrated better discriminative accuracy than the non-indexed parameters when differentiating between no or mild TR and moderate TR. Conversely, to differentiate moderate TR from severe TR, the AUC values of non-indexed parameters (TA area, TA major axis) were higher.

In our study, the analysis of ROC curves revealed that the highest AUC values were observed for TA area, perimeter, minor, and major axis in differentiating between moderate and severe TR. These findings are similar to the study conducted by A. Krivickiene et al., which also highlighted the predictive value of TA area and diameter parameters of the TV in identifying the severity of functional TR, specifically in distinguishing moderate from severe TR [17]. In contrast to our study, A. Krivickiene et al. investigated the changes of TV and RV geometry and function in various etiologies of ventricular functional TR (VF-TR) [17], whereas the majority of patients in our study had atrial functional TR (AF-TR).

These 3D parameters can be easily measured using TV dedicated software and implemented in daily practice for TA size assessment. Accurate TA sizing, comprehensive multiparametric TV, and TR evaluation may aid in decision-making regarding TR interventions. Certainly, future studies involving larger cohort are necessary to validate these threshold values.

### The phenotype of functional tricuspid regurgitation: AF-TR vs. VF-TR

The proposed functional TR classification follows the underlying TR etiology and TR mechanism: atrial functional TR (AF-TR) and ventricular functional TR (VF-TR) [7, 19]. From an echocardiographic standpoint, the AF-TR and VF-TR phenotypes are different. However, it is likely that in most patients, AF-TR and VF-TR phenotypes overlap in varying proportions [17, 20]. R. Hahn also discusses that in the advanced stage, VF-TR can evolve together with atrial fibrillation, and it can be challenging to distinguish the primary cause of TR [21]. In our study trying to classify TR according to functional TR mechanism, it was observed that it is not always possible to have a well-characterized phenotype for AF-TR or VF-TR. As there were some overlapping TR phenotypes, we decided to determine the AF-TR and non AF-TR groups (when the coexisting pathological conditions occurred). Emphasizing that in our study, non AF-TR is not equivalent to VF-TR, as this patient group comprises a mixture of etiologies.

The majority of the patients in our study had AF-TR. Additionally, there were more patients with severe TR in the AF-TR group. Similar to other studies [22, 23], we found that the patients in AF-TR group had larger TA area, perimeter, major axis, 4Ch diameter, and tenting volume compared to non AF-TR patients. However, the max tenting height, coaptation height, sphericity index, and FWS were similar between the AF-TR and non AF-TR patients. The parameters of TR severity (EROA and 3D VCA) had a stronger association with 3D TV metrics (TV tenting and TA area parameters) in AF-TR group.

These findings were supported by a study conducted by H. Utsunomiya et al., that investigated the differences in right heart remodeling and alterations in 3D TV geometry between AF-TR and VF-TR [23]. The analysis of 51 patients with severe TR, using QLAB mitral valve navigator software for TV measurements, revealed that the AF-TR group had more dilated TA, but less leaflet tethering, compared to the VF-TR group [23]. However, further analysis identified that patients with massive and torrential AF-TR had larger leaflet tethering and tenting volume compared to those with severe AF-TR. TA dilation accompanied by leaflet tethering is observed in advanced stage of AF-TR when TR is massive or torrential [23].

## Study limitations

There were several limitations in this study. Firstly, this was a single-center, cross-sectional study, and the study population was relatively small. Secondly, we did not evaluate TA dynamic changes throughout the cardiac cycle and with respiration. Thirdly, our analysis did not involve the 3D volumetric analysis of right heart chambers and the correlation between TA and other RV and RA parameters. Fourthly, we did not evaluate the effect of hemodynamic status and loading conditions on TR.

## Conclusion

Our study analyzes 3D TA metrics in normally functioning TV and in different grades of functional TR. There are gender-specific differences in TA dimensions, which disappear when indexed to BSA. 2D measurements are smaller than 3D. Three-dimensional TA parameters accurately differentiate between TR grades. Thus, we propose cut-off values of the 3D TA parameters in different functional TR grades.

### Supplementary Information

**Additional file 1: Table S1. **The parameters of tricuspid valve regurgitation and geometry according to TR phenotype.

## Data Availability

All data generated and analyzed in this study is available from the corresponding author on reasonable request.

## Abbreviations

AFI: Automated function imaging
BSA: Body surface area
Ch: Chambers
2D: Two-dimensional
3D: Three-dimensional
EROA: Effective regurgitant orifice area
FWS: Free wall longitudinal strain
MPR: Multiplanar reconstruction
MV: Mitral valve
PASP: Pulmonary artery systolic pressure
PISA: Proximal isovelocity surface area
RA: Right atrium
ROC: Receiver operating characteristic
RV: Right ventricle
TA: Tricuspid annulus
TAPSE: Tricuspid annular plane systolic excursion
TR: Tricuspid regurgitation
TV: Tricuspid valve
TVQ: Tricuspid valve quantification
VC: Vena contracta
