# Supplementary material for: Three-dimensional analysis of the tricuspid annular geometry in healthy subjects and in patients with different grades of functional tricuspid regurgitation
Source: Cardiovasc Ultrasound. 2023 Sep 15;21:17. doi: 10.1186/s12947-023-00315-7 (PMC10503068; doi:10.1186/s12947-023-00315-7)
Supplement: Supplementary file 1 — Additional file 1: Table S1. The parameters of tricuspid valve regurgitation and geometry according to TR phenotype. [file 12947_2023_315_MOESM1_ESM.docx]

**Table S1. The parameters of tricuspid valve regurgitation and geometry according to TR phenotype**

|  | **AF-TR**  **(n=63)** | **Non AF-TR**  **(n=32)** | **p-value** |
| --- | --- | --- | --- |
| **Sex (female)** | 37 (58.7) | 23 (71.9) | 0.209 |
| **Age, yrs** | 70.6 ± 12.3 | 65.9 ± 14.2 | 0.101 |
| **PASP, mmHg** | 38.7 ± 7.0 | 48.5 ± 17.0 | <0.001 |
| **VC width, cm** | 0.8 (0.5 – 1.2) | 0.5 (0.4 – 0.8) | 0.002 |
| **3D VCA, cm^2^** | 0.9 (0.5 – 1.6) | 0.4 (0.2 – 0.8) | <0.001 |
| **EROA, cm^2^** | 0.6 ± 0.3 | 0.4 ± 0.3 | 0.001 |
| **Regurgitant volume, ml** | 50.0 ± 20.1 | 35.8 ± 18.8 | 0.002 |
| **TR grade:**  **moderate**  **severe** | 24 (38.1)  39 (61.9) | 24 (75.0)  8 (25.0) | <0.001  <0.001 |
| **TA area, cm^2^** | 13.8 ± 3.5 | 11.1 ± 2.8 | <0.001 |
| **TA perimeter, cm** | 13.4 ± 1.7 | 12.0 ± 1.5 | <0.001 |
| **Major axis, cm** | 4.5 ± 0.6 | 4.1 ± 0.5 | <0.001 |
| **Minor axis, cm** | 3.7 ± 0.6 | 3.3 ± 0.5 | 0.282 |
| **4Ch diameter, cm** | 4.2 ± 0.6 | 3.6 ± 0.6 | <0.001 |
| **2Ch diameter, cm** | 3.7 ± 0.6 | 3.5 ± 0.6 | 0.109 |
| **Max tenting height, cm** | 0.8 ± 0.3 | 0.7 ± 0.3 | 0.067 |
| **Coaptation height, cm** | 0.7 ± 0.4 | 0.6 ± 0.2 | 0.079 |
| **Tenting volume, ml** | 2.8 (1.6 – 4.2) | 1.6 (0.7 – 2.8) | 0.005 |
| **Sphericity index, %** | 82.0 ± 9.1 | 80.2 ± 9.9 | 0.367 |
| **FWS, %** | -20.2 ± 5.4 | -20.9 ± 7.3 | 0.569 |
| **TAPSE, cm** | 1.3 ± 0.3 | 1.6 ± 0.7 | 0.033 |

Values are mean ± SD; median ﻿(interquartile range) or n (%);

AF-TR – atrial functional tricuspid regurgitation; 4Ch – four chambers; EROA – effective regurgitant orifice area; FWS – free wall strain; PASP – pulmonary artery systolic pressure; 3D – three dimensional; TA – tricuspid annulus; TAPSE – tricuspid annular plane systolic excursion; TR – tricuspid regurgitation; 2Ch – two chambers; VC – vena contracta; VCA – vena contracta area.
